# Generative AI with WGAN-GP for boosting seizure detection accuracy

**DOI:** 10.3389/frai.2024.1437315

**Published:** 2024-10-02

**Authors:** Lina Abou-Abbas, Khadidja Henni, Imene Jemal, Neila Mezghani

**Affiliations:** ^1^Applied Artificial Intelligence Institute (I2A), TELUQ University, Montreal, QC, Canada; ^2^Department of Science and Technology, TELUQ University, Montreal, QC, Canada; ^3^Department of Electrical and Computer Engineering, Lebanese American University, Byblos, Lebanon

**Keywords:** epileptic seizure, EEG, data augmentation, WGAN-GP, LSTM

## Abstract

**Background:**

Imbalanced datasets pose challenges for developing accurate seizure detection systems based on electroencephalogram (EEG) data. Generative AI techniques may help augment minority class data to facilitate automatic epileptic seizure detection.

**New method:**

This study investigates the impact of various data augmentation (DA) approaches, including Wasserstein Generative Adversarial Network with Gradient Penalty (WGAN-GP), Vanilla GAN, Conditional GAN (CGAN), and Cramer GAN, on classification performance with Random Forest models. The best-performing GAN variant, WGAN-GP, was then integrated with a bidirectional Long Short-Term Memory (LSTM) architecture and compared against traditional and synthetic oversampling methods.

**Results:**

The evaluation of different GAN variants for data augmentation with Random Forest classifiers identified WGAN-GP as the most effective approach. The integration of WGAN-GP with bidirectional LSTM yielded substantial performance improvements, outperforming traditional oversampling methods and achieving an accuracy of 91.73% on the augmented data, compared to 86% accuracy on real data without augmentation.

**Comparison with existing methods:**

The proposed generative AI approach combining WGAN-GP and recurrent neural network models outperforms comparative synthetic oversampling methods on metrics relevant for reliable seizure detection from imbalanced EEG datasets.

**Conclusions:**

Incorporating the WGAN-GP generative AI technique for data augmentation and integrating it with bidirectional LSTM elevates seizure detection accuracy for imbalanced EEG datasets, surpassing the performance of traditional oversampling and class weight adjustment methods. This approach shows promise for improving epilepsy monitoring and management through enhanced automated detection system effectiveness.

## Highlights

- Epilepsy detection systems often struggle with imbalanced datasets, impacting their accuracy.
- This study explores the potential of generative AI techniques for augmenting EEG data to facilitate improved seizure detection.
- The proposed WGAN-GP data augmentation approach, when integrated with a bidirectional LSTM architecture, outperforms conventional synthetic data generation methods for boosting seizure detection performance.

## 1 Introduction

Epilepsy is a neurological condition characterized by recurring seizures, abrupt disruptions in the brain's usual electrical activity (Fisher et al., 2005). These seizures can manifest in various symptoms, including muscle spasms, loss of consciousness, and alterations in behavior. Timely identification of an impending seizure empowers individuals to mitigate its impact by seeking medical assistance (World Health Organization, 2006).

Electroencephalography (EEG) serves as a widely employed diagnostic tool in epilepsy, playing a vital role in assessing treatment options for seizure management (Stafstrom and Carmant, 2015; Jemal et al., 2022). However, the practical implementation of EEG-based systems for seizure detection and prediction encounters significant hurdles, such as protracted calibration time and limited applicability. The primary challenge in seizure research lies in collecting data during actual seizure occurrences, primarily due to their unpredictable nature. Acquiring an extensive and diverse sample size required for obtaining reliable outcomes poses a substantial obstacle (Kuhlmann et al., 2018).

Imbalanced data in the context of data analysis refers to scenarios where the quantity of data points within one class significantly differs from another class. This imbalance can impede the accuracy of machine learning models in predicting the occurrence of epilepsy, as models can be more influenced by the more prevalent class, typically the negative examples (Kuhlmann et al., 2018). Consequently, this leads to subpar performance and increased false negatives, affecting the precision and reliability of machine learning models in predicting seizure outcomes or classifying different types of seizures.

Addressing the challenge of imbalanced data is crucial for advancing epilepsy research and treatment. Several approaches can be employed, including resampling techniques like oversampling or undersampling, and algorithms specifically designed for imbalanced data, such as decision trees or random forests.

Data augmentation is another valuable technique, which involves generating new synthetic examples based on existing data. Various methods for data augmentation include synthetic data generation, data transformation, data combination, and data noise injection (Dubey et al., 2014; Johnson and Khoshgoftaar, 2019; Kraiem et al., 2021). By using these techniques, it may be possible to overcome the challenge of imbalanced data and enhance the performance of machine learning models in epilepsy research. It is also important to carefully evaluate the performance of the model on the imbalanced data, using metrics such as precision, recall, and the F1 score, rather than solely relying on overall accuracy.

The primary objective of this investigation was to examine various data augmentation (DA) approaches in order to tackle the issue of imbalanced data. To amplify the representation of the minority classes, we explored diverse generative AI techniques. Initially, we employed four different variants of deep learning generative adversarial networks (GAN) to generate synthetic data targeting the underrepresented classes. We conducted a projection analysis and Qq plot analysis to compare the synthetic and real data and ascertain the GAN variant that accurately replicated the real data.

Furthermore, we utilized conventional DA methods such as SMOTE and ADASYN to expand the existing dataset. We evaluated the effectiveness of these techniques in comparison to the most effective GAN variant. To analyze the impact of data augmentation (DA) techniques on classification performance, we trained Long Short-Term Memory (LSTM) models and evaluated their performance on datasets with and without DA. Similarly, we used the Random Forest algorithm to compare performance metrics between classifiers trained solely on real data and those trained on a combination of real and synthetic data generated by DA techniques. This comparison helps us understand how different DA methods influence the LSTM and RF's classification accuracy and other performance metrics.

Our primary contribution lies in the systematic evaluation of various GAN architectures and conventional oversampling techniques for generative data augmentation in order to address class imbalance in seizure detection using LSTM classifiers. We demonstrate the effectiveness of WGAN-GP in synthesizing minority class data that closely aligns with the distribution of real data, resulting in significant performance improvement. This provides a crucial framework and benchmark for further research on data augmentation strategies for managing imbalanced data in EEG classifications and seizure detection.

## 2 Related state of art

In recent years, DA techniques have emerged as a promising approach to improve the performance of machine learning models in epilepsy detection tasks. By increasing the amount of available training data, DA can help to reduce overfitting and improve the generalization performance specifically when dealing with tasks based on electroencephalograms (EEGs).

For tasks such as emotion recognition (Wang et al., 2018; Salama et al., 2018; Luo and Lu, 2018; Chang and Jun, 2019), DA techniques have been shown to be instrumental in improving the performance of learning models. In the field of seizure detection, numerous investigations have employed DA techniques to advance the accuracy of deep learning models (Wei et al., 2019; Hussein et al., 2018).

A comprehensive review conducted by Lashgari et al. (2020) explored different DA approaches such as noise addition, GAN networks, sliding windows, sampling, Fourier transform, and recombination of segmentation and showed that DA is becoming a prevalent approach in EEG tasks, resulting in a significant improvement in accuracy, with an average increase of 29%.

The method proposed by Yuan et al. (2017), using the weighted extreme learning machine (ELM), showed to be an effective approach to mitigate the impact of the imbalanced class distribution on performance.

In a new study published in 2022, Zhang et al. (2022), a new algorithm called BNNSMOTE was proposed to improve seizure detection performance by synthesizing new samples to address the challenge of imbalanced classification of seizure and non-seizure data.

In other studies (Ullah et al., 2018; Wang et al., 2021), augmentation techniques were used to address the problem of limited data and to improve the performance of the one-dimensional convolutional neural network (P-1D-CNN) model in the detection of seizures. Learned models showed high accuracy, sensitivity, and specificity.

Furthermore, researchers proposed using synthetic data generated by Temporal GAN to increase sample size and improve deep learning classification performance when faced with the limited availability of labeled thalamic EEG data (Ganti et al., 2022).

In addition, Zhao et al. (2022) presented an EEG augmentation method (EEGAug), involving the random selection of a few samples from the underrepresented class, the transformation of these samples into the frequency domain, the combination of different frequency bands, and conversion back to the time domain to generate new samples. Through the use of EEGAug, the authors balanced the unbalanced clinical iEEG data, which performed the best in most cases. Three studies have proposed using GANs for DA in different applications.

In one study, a WGAN-GP model is proposed for DA in emotion recognition using the DEAP dataset (Bhat and Hortal, 2021).

In another study (Hartmann et al., 2018), GANs are used to generate electroencephalographic (EEG) brain signals. A modification to the training of Wasserstein GANs is introduced to stabilize the training process.

In the third study (Haradal et al., 2018), a new method is presented to create synthetic time series data using GANs for the classification of biosignals. These approaches can have limitations and may not always be effective.

Based on the current state of the art in epilepsy detection, it is clear that DA and the issue of imbalanced datasets need to be further addressed. While recent studies have shown promising results using GANs for DA in EEG-based classification tasks, more research is needed to explore the potential of these techniques for improving the accuracy and reliability of epileptic seizure detection. As such, there is a pressing need for more studies to focus on developing effective strategies for DA and addressing data-imbalanced issues in epilepsy detection.

## 3 Method and materials

### 3.1 Database

The current research utilized data obtained from the Temple University Hospital EEG Seizure Corpus (TUSZ), version v1.5.1. This corpus consists of EEG recordings obtained in real-time from 341 patients undergoing clinical monitoring. The recordings were conducted utilizing the 10/20 international standard system, and among these patients, 188 were female. It includes 886 sessions, each ranging from one minute to one hour, thus providing a varied and diverse array of EEG signals. The TUSZ includes approximately 6% of files containing EEG seizure segments, which amount to a total duration of 40.41 hours. The TUSZ dataset offers several montages, but for consistency in this study, we focused exclusively on the most popular bipolar Temporal Central Parasagittal (TCP) Averaged Reference (AR) montage, ensuring uniformity across all analyzed data. All EEG signals were sampled at a minimum rate of 250 Hz to ensure recording accuracy. Prior to analysis, qualified researchers removed any artifacts and eye blinks present in the recordings by using an open-source annotation tool. Table 1 provides detailed information on the key features of the corpus used in the study. The corpus was carefully selected for its diverse range of EEG signals, providing an opportunity to conduct an in-depth analysis of seizure segments in a clinical setting. For more information about the database used, we invite readers to read Shah et al. (2018).

**Table 1:** Detailed overview of the Temple University Hospital EEG Seizure Corpus (TUSZ) used in our seizure detection experiments.

**Number of patients (female)**	**341 (188 F)**
Number of patients with seizure	133 (72 F)
Total number of sessions	886
Total number of files	7,634
Number of seizure files	1,780
Number of seizure-free files	5,854
Total duration in hrs.	655.36

### 3.2 The proposed framework

**Figure 1:**
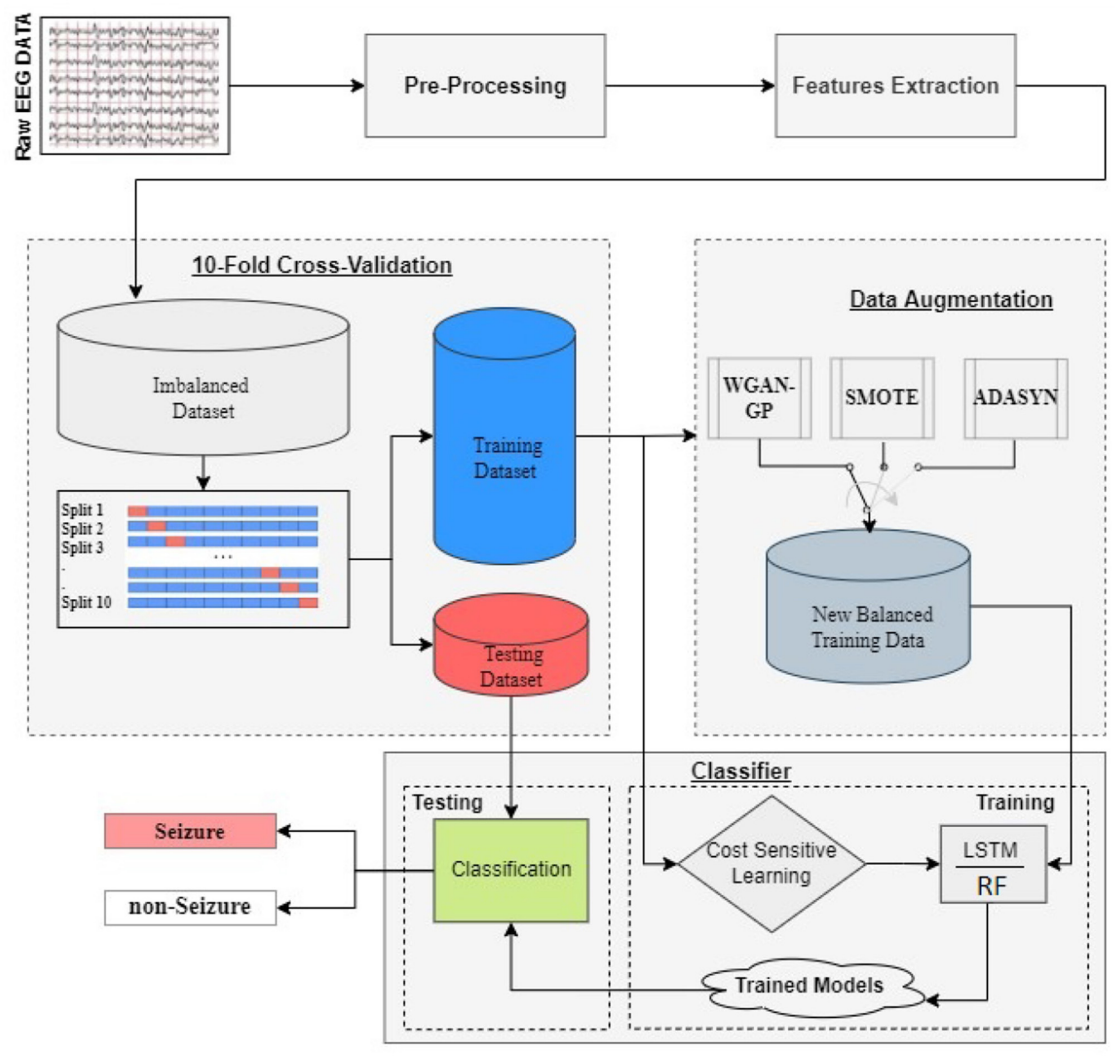
Framework for seizure detection and classification using DA strategies to address imbalanced EEG signal datasets: a proposed methodology utilizing 10-fold cross-validation and LSTM/RF models for training and testing.

Figure 1 displays the proposed framework for detecting and classifying seizures using DA techniques to address imbalanced datasets. The framework consists of two primary steps. In the first step, pre-processing of the EEG signal is performed, followed by feature extraction. For each of the 19 EEG channels, 44 time and/or frequency domain features are extracted, resulting in a total of 836 features. The list of features is detailed in Table 2 and for more details refer to our previous work (Abou-Abbas et al., 2023, 2022, 2021).

**Table 2:** List of all the 44 features extracted.

**Number**	**Features**
1–5	Average value of power spectral density (delta, theta, alpha, beta, gamma)
6–10	Absolute value of power spectral density (delta, theta, alpha, beta, gamma)
11–15	Relative value of power spectral density (delta, theta, alpha, beta, gamma)
16–18	Skewness, variance and kurtosis
19–24	Features of empirical mode decomposition (energy, spectral entropy, mean, standard deviation, moment, skewness)
25–29	Sample entropy, permutation entropy (4 levels)
30–32	Hjorth (mobility, activity, complexity)
33	Spectral entropy of PSD
34	Features of discrete wavelet transform-Shannon entropy
35–36	Features of wavelet packet decomposition (log energy entropy and Shannon entropy)
37	Successive decomposition index
38–39	Mean Energy and its cumulative sum
40–44	Features of wavelet Decomposition (Percentage of energy-5 levels)

Next, a 10-fold cross-validation method is employed to partition the data. The cross-validation is performed using the GroupKFold technique, to ensure that all samples from a single patient are either included entirely in the training set or entirely in the test set for each fold. This prevents any overlap of patient information between the training and test phases. Specifically, the data is divided into 10 groups (or folds), and for each iteration, 9 of these folds are used to train the classifier, while the remaining fold is used for testing. This process is repeated ten times, with each fold serving as the test set once. The performance measures are averaged across all ten iterations to obtain the final results.

The training set is then utilized in two distinct ways. The first approach employs DA techniques, such as WGAN-GP, SMOTE, or ADASYN, to balance the dataset. The newly balanced dataset is then used to train the LSTM model. The second approach directly trains the LSTM model using the imbalanced dataset, but with a cost-sensitive loss function or class weighting approach. In both scenarios, the test data is reserved for testing the trained model.

### 3.3 Data augmentation

DA is a technique utilized to create new training data by altering the existing data. Its primary goal is to enhance the performance of classification systems by forming new and different examples to train datasets. It is a valuable tool in situations where collecting new data is difficult or costly (Mumuni and Mumuni, 2022). It can be achieved by making small changes to the dataset or using deep learning models to generate new data points to expand the training set, enhance its diversity, and improve model generalization. Generative DA techniques, such as GANs and their variants like CramerGAN, CGAN, Vanilla GAN, and WGAN-GP, employ generative models to produce entirely new data points that resemble the real data distribution (Goodfellow et al., 2020; Creswell et al., 2018; Gulrajani et al., 2017; Arjovsky et al., 2017). These generative approaches enable the creation of realistic synthetic samples that capture the underlying characteristics of the training data. On the other hand, synthetic DA techniques focus on creating new samples by applying transformations or modifying existing data points. This includes techniques like SMOTE and ADASYN, which generate synthetic samples for minority classes by interpolating between existing instances or adapting the generation process based on the data distribution (Chawla et al., 2002; He et al., 2008). Several studies have explored DA techniques in different fields. For instance, a study proposed a general methodology for small medical data classification that deploys an augmentation technique and a feature selection strategy (Alauthman et al., 2023). Another study presented a comprehensive survey of modern DA techniques (Mumuni and Mumuni, 2022). In this study, we will explore the generation of data using GAN (Generative Adversarial Network) and its variants, including CramerGAN, CGAN, Vanilla GAN, and WGAN-GP. Additionally, we will compare these generative approaches with synthetic data generation techniques such as SMOTE and ADASYN. Through comparative analysis with synthetic approaches like SMOTE and ADASYN, we will assess the effectiveness of generative DA in addressing class imbalance and improving the performance of machine learning models.

#### 3.3.1 Generative Adversarial Network and its variants

**Figure 2:**
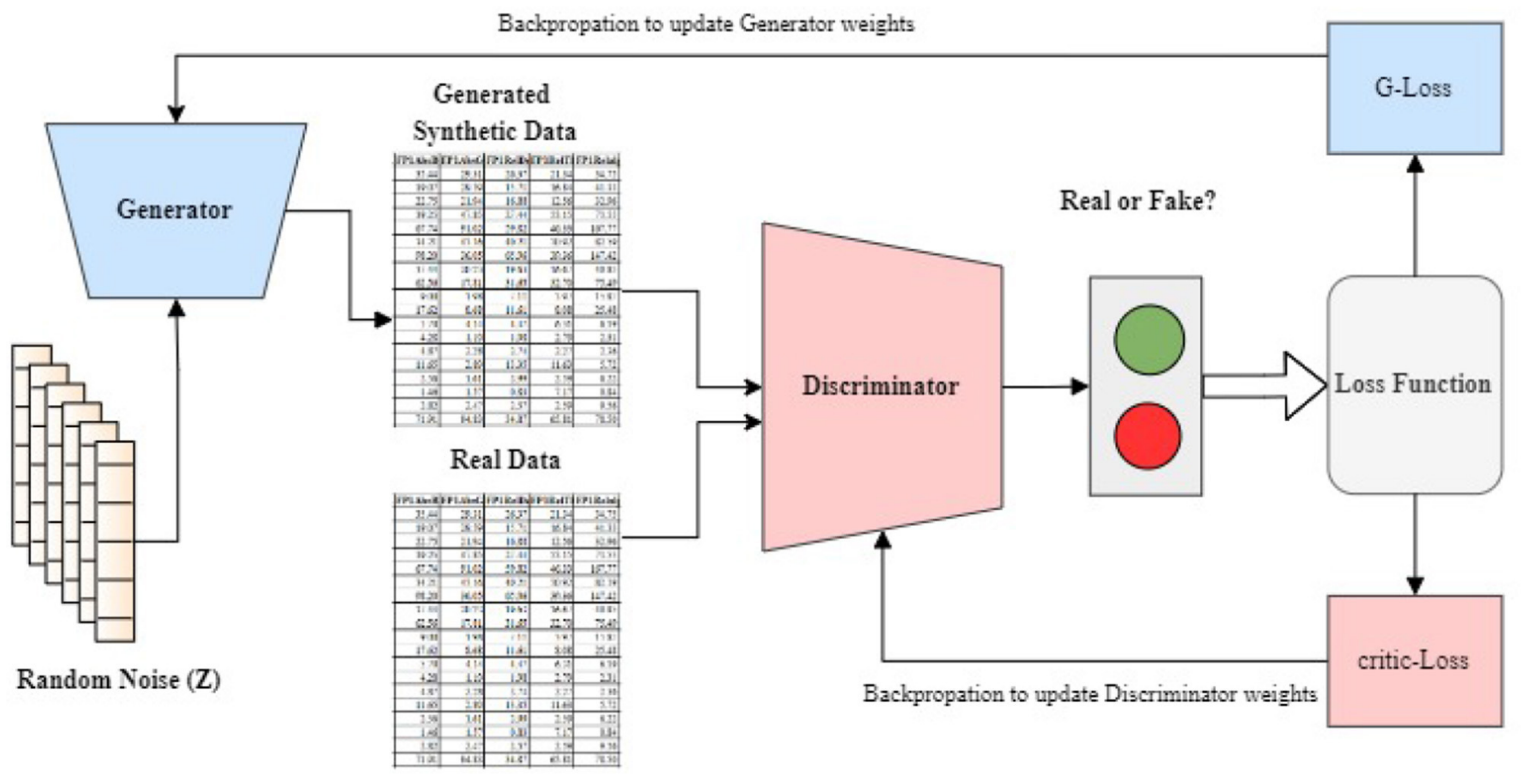
GAN architecture with critic loss and backpropagation: updating discriminator and generator weights to improve signal generation.

Generative Adversarial Networks, or GANs for short, have become a very powerful deep learning technique for data generation and augmentation. GANs are made up of two key components - a generator and a discriminator. They work by having the generator creates synthetic data, while the discriminator evaluates this data to determine whether it is real or artificial. The original GAN architecture, introduced by Goodfellow et al. (2020) and Creswell et al. (2018) is commonly referred to as the Vanilla GAN. Over the years, researchers have proposed numerous GAN variants to address various challenges and limitations to the original formulation to improve performance and extend their applications (Gulrajani et al., 2017; Arjovsky et al., 2017; Bellemare et al., 2017; Mirza and Osindero, 2014; Gulrajani et al., 2017). One notable variant, the CramerGAN, replaces the discriminator with a Cramer distance estimator improving training stability and the quality of generated samples (Bellemare et al., 2017). Another influential variant is the Conditional GAN (CGAN), which incorporates additional conditioning information such as class labels or auxiliary features, into both the generator and discriminator networks to enable better control over the generated data (Mirza and Osindero, 2014). The Wasserstein GAN with Gradient penalty (WGAN-GP) proposed by Gulrajani et al. (2017) and Arjovsky et al. (2017) addresses training instability and mode collapse issues by incorporating the Wasserstein distance as a more meaningful loss function. The gradient penalty further enhances training stability, encouraging the discriminator to behave as a true Wasserstein distance estimator. Figure 2 gives a detailed architecture of a basic GAN. A random noise (z) is used as input to the generator, a typical feature of GANs. The discriminator evaluates both real data from the dataset and the synthetic data generated. It assigns probabilities between 0 and 1 to these data samples, indicating their likelihood of being real or fake. The critic loss also called discriminator's loss is computed based on how well its predictions match the ground truth labels from the training dataset. This creates a feedback loop where the generator and discriminator continuously adjust: the generator improves the quality of its synthetic data to reduce its loss, while the discriminator enhances its ability to distinguish between real and synthetic data to minimize its own loss.

#### 3.3.2 Synthetic data generation techniques: SMOTE and ADASYN

The Synthetic Minority Over-sampling Technique (SMOTE) is a widely used method for addressing the issue of imbalanced datasets. SMOTE works by creating new samples for the minority class in a dataset. For each existing sample in the minority class, it finds a few similar samples, called its nearest neighbors. It then creates new samples by mixing the original sample with these neighbors. To do this mixing, SMOTE uses a simple math formula. For each minority class sample *x*_*i*_, SMOTE selects *k* nearest neighbors. Specifically, if *x*_*i*_ and *x*_*j*_ are two samples from the minority class, a new synthetic sample *x*_*new*_ is generated using:

*x*_*new*_ = *x*_*i*_+Λ.(*x*_*j*_−*x*_*i*_) where Λ is a random number between 0 and 1. The algorithm repeats this process until the desired number of synthetic samples is generated. By creating these new samples, SMOTE expands the decision region of the minority class, helping to balance the dataset and potentially improving the performance of classification algorithms on the minority class (Chawla et al., 2002).

The Adaptive Synthetic Sampling (ADASYN), is another DA technique that generates synthetic samples of the underrepresented class. However, ADASYN diverges from other techniques by employing a distinct approach wherein the density distribution of the minority class is estimated and then the synthetic samples are produced in regions characterized by low density. Mathematically, ADASYN operates as follows: For each minority sample *x*_*i*_, the density ratio *r*_*i*_ is calculated as:


(1) $$\begin{array}{l} r_{i}=\frac{\Delta_{i}}{K} \end{array}$$

where Δ_*i*_ represents the number of majority class samples among its *K* nearest neighbors. These ratios are then normalized to form a distribution:


(2) $$\begin{array}{l} {\hat{r}}_{i}=\frac{r_{i}}{\sum r_{i}} \end{array}$$

The number of synthetic samples *g*_*i*_ to generate for each *x*_*i*_ is determined by:


(3) $$\begin{array}{l} g_{i}=G\cdot {\hat{r}}_{i} \end{array}$$

where *G* is the total number of synthetic samples needed. New synthetic samples are created using linear interpolation:


(4) $$\begin{array}{l} x_{new}=x_{i}+\lambda \cdot \left(x_{j}-x_{i}\right) \end{array}$$

where *x*_*j*_ is a randomly chosen minority neighbor of *x*_*i*_, and λ is a random number in the interval [0, 1]. This process is repeated *g*_*i*_ times for each *x*_*i*_, focusing on generating synthetic samples in regions where the minority class is underrepresented, thus adapting to the local data distribution (He et al., 2008).

### 3.4 LSTM architecture

Long Short-Term Memory (LSTM) networks are a special type of neural network designed to handle sequential data, like EEG signals. They're good at remembering important information over long periods, which is crucial for tasks like seizure detection. At its heart, an LSTM has a cell state, a sort of memory, and three principal components as gates: the input gate, which decides what new information to add to the cell state; the forget gate, which chooses what information to remove from the cell state; and the output gate, which determines what to output based on the cell state. The combination of all these gates provides LSTMs with the ability to selectively remember or forget information over long sequences, making them really powerful in many tasks related to data that comes in a sequence or time series, such as EEG signal analysis data (Hochreiter and Schmidhuber, 1997).

**Figure 3:**
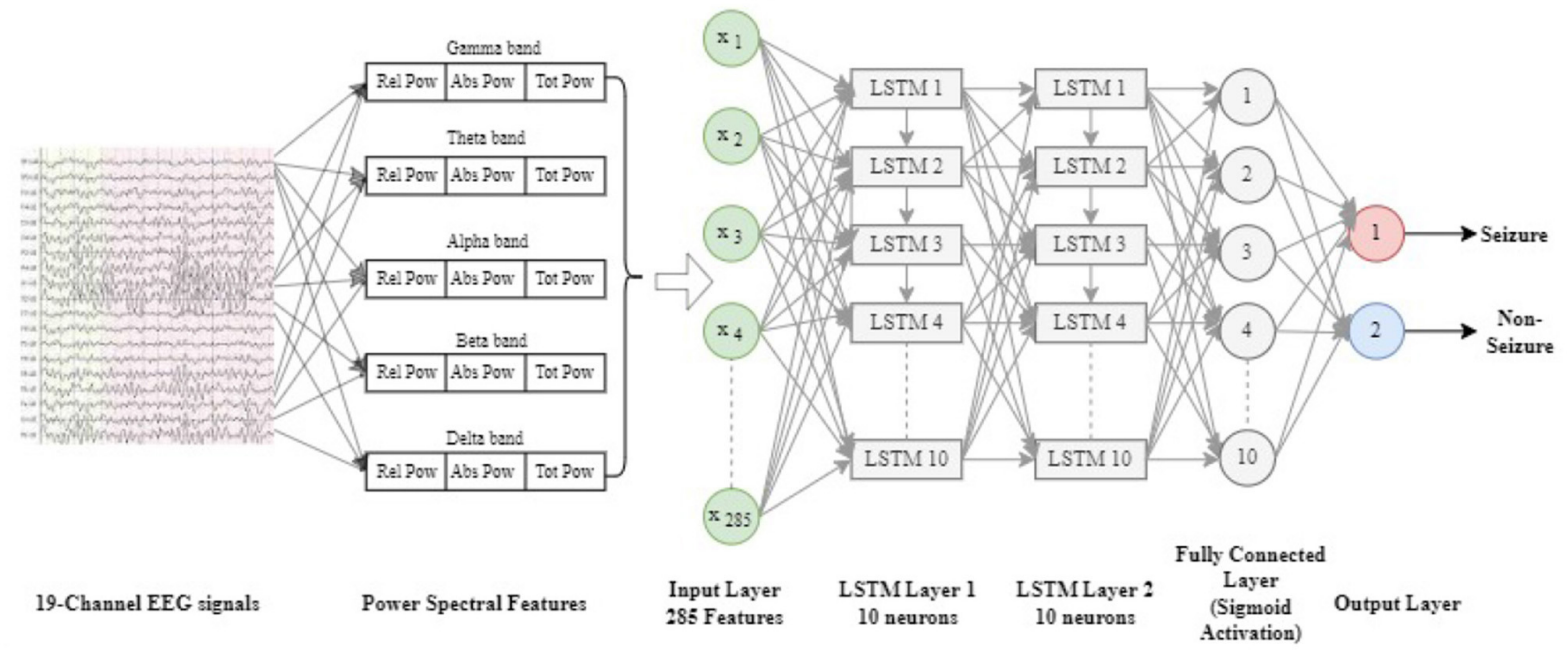
LAST architecture used for classification of raw EEG data for seizure detection.

Figure 3 illustrates the LSTM architecture implemented for classifying raw EEG data to detect seizures. This architecture is designed to leverage the strengths of LSTMs in processing sequential data. The LSTM network used in this study is unidirectional, meaning it processes data in a single direction, from past to future, which is appropriate for the nature of sequential EEG data.

The input layer of the network receives 836 features derived from the raw EEG signals. These features are fed into two LSTM layers, each containing 10 neurons. Each LSTM unit within these layers applies the three gates–input, forget, and output gates–to manage the flow of information and maintain memory across time steps.

Following the LSTM layers, the network includes a fully connected (dense) layer. This layer transforms the output from the LSTM layers into a one-dimensional vector, which is then used for classification. The final output is passed through a Sigmoid activation function, which produces a probability score between 0 and 1, indicating the likelihood of a seizure event.

## 4 Results and discussion

### 4.1 Experimental setup

The framework illustrated in Figure 1 provides a comprehensive overview of the methodology. It starts with data augmentation (DA) using various Generative Adversarial Networks (GANs) and other DA techniques, including Vanilla GAN, CRAMER GAN, CGAN, WGAN-GP, SMOTE, and ADASYN. These methods were selected due to their ability to learn and generate realistic data distributions, enhancing the representation of minority classes in the augmented dataset. Once the augmented dataset is created, it is split into training and testing sets using a 10-fold cross-validation approach to ensure robust performance evaluation.

To evaluate the effectiveness of the DA approaches, the study employs several assessment methods. Initially, Quantile-Quantile (QQ) plot analysis is conducted to compare and analyze the distribution of real and synthetic data, providing insights into the fidelity of the synthetic data generation process and its alignment with the real data distribution. Additionally, projection techniques such as Fast Independent Component Analysis (Fast ICA), Kernel Principal Component Analysis (Kernel PCA), Truncated Singular Value Decomposition (Truncated SVD), and t-Distributed Stochastic Neighbor Embedding (t-SNE) are utilized to analyze the results and evaluate the performance of the DA techniques.

The core of the study's experimental setup focuses on training and evaluating machine learning models using the augmented dataset. Both Random Forest (RF) and Long Short-Term Memory (LSTM) models are employed. The RF algorithm serves as a preliminary benchmark in our study, assessing the classifier's performance when trained on both real data and a mix of real and synthetic data. This evaluation is essential for understanding how data augmentation impacts a traditional machine learning model. Our previous work Abou-Abbas et al. (2022), has established that RF significantly outperforms other standard classifiers, including Support Vector Machines (SVM). Following this preliminary evaluation, the LSTM model, tailored for handling the sequential characteristics of EEG signals, is then trained and assessed. This approach allows us to explore how more advanced deep learning techniques compare with RF in seizure detection.

TensorFlow and Keras were used to implement the DA techniques, LSTM models, and cross-validation schemes (Fabio et al., 2013). The Adam optimizer as a gradient-based method with with β_1_ = 0.9, β_2_ = 0.999 and a learning rate of 0.001 was employed for model training. We used the early stopping criteria to prevent over-fitting where training runs up to 100 epochs, or until the validation loss does not decrease anymore for at least 20 epochs.

Several tests are performed to assess the effectiveness of each DA technique on the Temple University Seizure Detection Corpus (TUSZ) data. The experiments are repeated 10 times using different subsets of the dataset to ensure robust results. The performance of the LSTM model is compared across various DA techniques, providing a comprehensive analysis of how each method impacts the classification of imbalanced EEG signal datasets.

### 4.2 Comparative evaluation of GAN variants

A thorough comparative evaluation was conducted to assess the performance of different GAN variants in generating synthetic data that closely resembled real data distributions. The evaluation process involved analyzing the Q-Q plots which compare two sets of data by plotting their quantiles against each other. If the synthetic data matches the real data well, the points on the plot will form a nearly straight line, usually at a 45-degree angle. This line would mean the two sets of data have very similar distributions. When points stray from this line, it shows differences between the synthetic and real data. It also helps us judge if our methods for creating synthetic data are effectively balancing out our dataset, which originally had uneven amounts of data in different categories.

**Figure 4:**
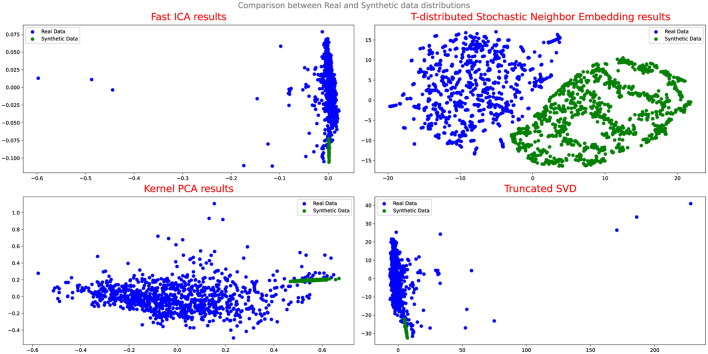
Comparison between real and synthetic data distribution vanilla GAN.

**Figure 5:**
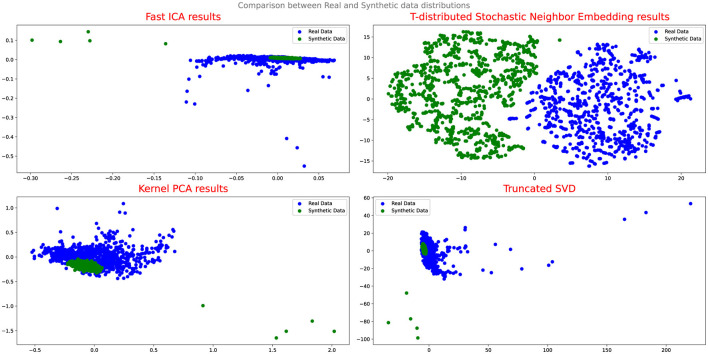
Comparison between real and synthetic data distribution-CGAN.

Figures 4, 5 illustrate the results of the projection analysis, specifically for the synthetic data generated respectively using Vanilla GAN and CGAN. As depicted in the figures, the projected synthetic data appears to be clustered together, significantly deviating from the distribution of the real data. These observation suggests that the synthetic data generated by Vanilla GAN and CGAN fails to capture the inherent patterns and structure present in the real data distribution. Moreover, the form and shape of the synthetic data in Figures 4, 5 differ noticeably from that of the real data, indicating a significant disparity between the two distributions.

**Figure 6:**
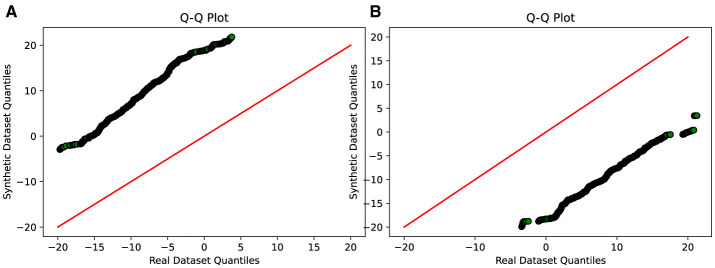
Q-Q plot analysis-visual representation of the similarity between the synthetic and real data distributions. **(A)** Comparison between real and synthetic data generated using VanillaGAN. **(B)** Comparison between real and synthetic data generated using CGAN.

Furthermore, the Q-Q plot analysis reinforces these findings. The Q-Q plot in Figures 6A, B depicts a notable misalignment between the synthetic and real data distributions, with the points deviating considerably from the ideal diagonal line. This misalignment further supports the notion that Vanilla GAN and CGAN struggle to generate synthetic data that closely resembles the distribution of the real data.

**Figure 7:**
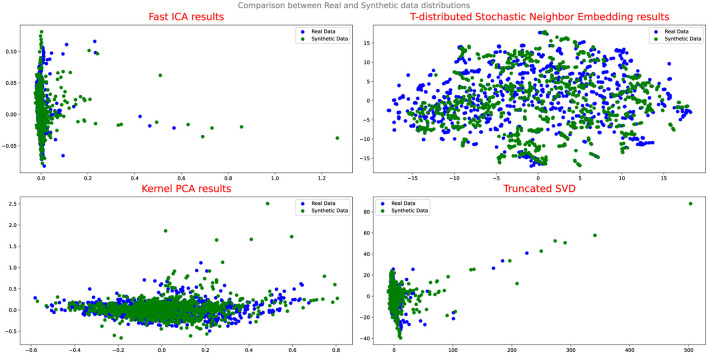
Comparison between real and synthetic data distribution-CRAMERGAN.

Upon evaluating the results of the projection analysis and Q-Q plots for Cramer GAN, it is observed that its performance is slightly improved compared to Vanilla GAN. Figure 7 displays the projection results of the synthetic data generated by Cramer GAN, where the dispersion of the synthetic data points appears to be more aligned with the real data distribution.

**Figure 8:**
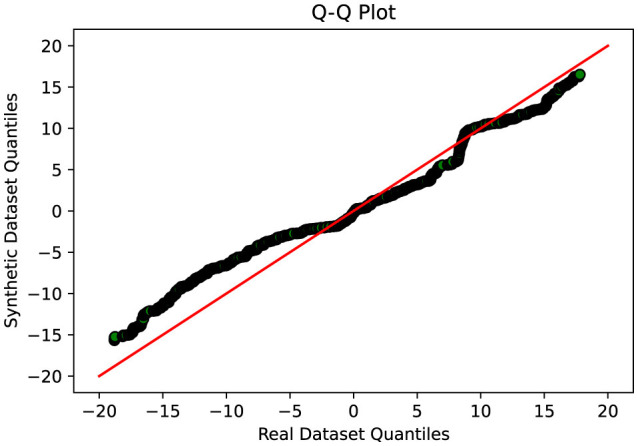
Q-Q plot analysis-visual representation of the similarity between the synthetic and real data distributions-CRAMERGAN.

**Figure 9:**
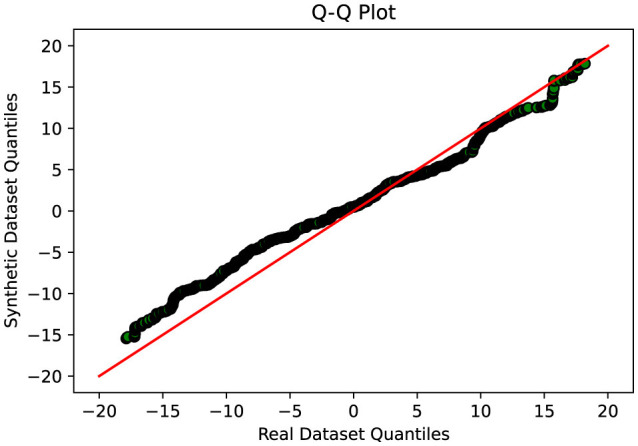
Q-Q plot analysis-visual representation of the similarity between the synthetic and real data distributions-WGAN-GP.

**Figure 10:**
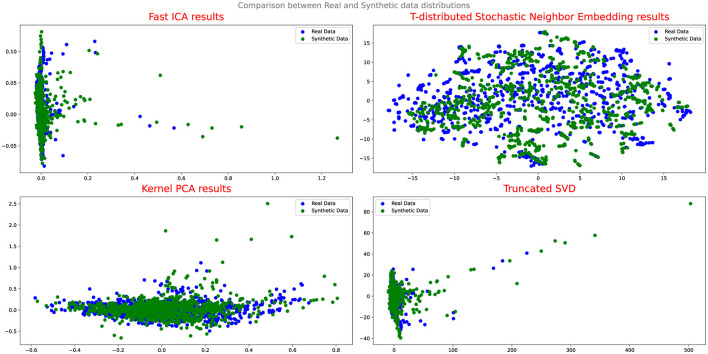
Comparison between real and synthetic data distribution WGAN-GP.

This indicates that Cramer GAN has a better ability to capture the underlying structure and patterns of the real data, resulting in a more coherent and representative synthetic data distribution. Moreover, the Q-Q plot analysis in Figure 8 reveals a closer alignment between the synthetic and real data distributions, as the points in the plot exhibit a higher degree of adherence to the ideal diagonal line in the Q-Q plot. This suggests that CramerGAN generates synthetic data that closely mirrors the statistical characteristics of the real data. In both Q-Q plots and projection analyses, CramerGAN outperforms Vanilla GAN and CGAN. However, when comparing CramerGAN to WGAN-GP, the Q-Q plot results are quite similar as seen in Figure 9. The difference becomes visually apparent in the projection results shown in Figure 10, where WGAN-GP demonstrates superior performance. The synthetic data points generated by WGAN-GP show a remarkable alignment and integration with the real data, indicating a high degree of similarity and coherence. This enhanced performance suggests that WGAN-GP excels at capturing the underlying structure and patterns of the real data, resulting in a synthetic data distribution that closely resembles the real data distribution. The substantial improvements achieved by WGAN-GP in the projection analysis emphasize its efficacy in generating synthetic data that exhibits a remarkable resemblance to the real data distribution.

The substantial improvements achieved by WGAN-GP in both projection analysis and Q-Q plots emphasize its efficacy in generating synthetic data that exhibits a remarkable resemblance to the real data distribution.

### 4.3 Impact of data augmentation on classification performance with random forest

The Impact of DA on Classification Performance with Random Forest was investigated, and the results are summarized in Table 1. The table compares the classification performance metrics for two scenarios: using real data only and using a combination of real and synthetic data. The metrics considered are Accuracy, Recall, Specificity, F1 Score, and Precision. The results in Table 3 demonstrate the impact of augmenting the data and training the Random Forest model with a combination of real and synthetic data to balance the dataset. By incorporating synthetic data, the overall performance of the classification model improved. The Accuracy of the model increased from 0.86 when using real data only to 0.88 when utilizing real and synthetic data. Similarly, the Recall increased from 0.79 to 0.83, indicating a higher ability to correctly identify positive instances.

**Table 3:** Classification performance metrics for real data only and real + synthetic data.

**Metrics**	**Real data only**	**Real + synthetic data**
Accuracy	0.86	0.88
Recall	0.79	0.83
Specificity	0.67	0.72
F1 Score	0.83	0.86
Precision	0.81	0.84

Furthermore, the Specificity, which represents the model's ability to correctly identify negative instances, also improved from 0.67 to 0.72. The F1 Score, a harmonic mean of precision and recall, increased from 0.83 to 0.86, indicating a more balanced performance between these two metrics. Lastly, the Precision increased from 0.81 to 0.84, demonstrating the model's improved ability to correctly classify positive instances.

### 4.4 Classification for seizure detection using bi-directional LSTM

Table 4 presents the classification performance metrics for different DA approaches in the task of seizure detection. Four approaches were evaluated based on various metrics including Loss, Precision, Recall, Specificity, and Accuracy. In terms of Loss, the SMOTE approach had a value of 0.1252, followed by ADASYN with 0.1281, Class Weight with 0.1255, and WGAN-GP with the lowest value of 0.0681. A lower loss value indicates better model performance in minimizing the discrepancy between predicted and actual values. Regarding Precision, the WGAN-GP approach achieved the highest precision of 80.81%. This indicates that it had a higher proportion of correctly classified positive instances compared to the other approaches. ADASYN had the second highest precision at 61.50%, followed by SMOTE with 60.38%, and Class Weight with the lowest precision of 63.03%. For Recall, the WGAN-GP approach achieved a value of 76.71%, indicating its ability to correctly identify positive instances. ADASYN had the second highest recall at 71.91%, followed by Class Weight with 72.93%, and SMOTE with the lowest recall of 70.57%. The WGAN-GP approach obtained the highest specificity of 95.45%, indicating its ability to distinguish negative instances accurately. ADASYN achieved the second highest specificity at 88.47%, followed by Class Weight with 87.76%, and SMOTE with the lowest specificity of 88.90%.Moreover, the WGAN-GP approach achieved the highest accuracy of 91.73%, followed by ADASYN at 85.18%, Class Weight at 84.82%, and SMOTE with the lowest accuracy of 85.25%.

**Table 4:** Classification performance metrics for seizure detection.

**Approach**	**Loss**	**Precision**	**Recall**	**Specificity**	**Accuracy**
SMOTE	0.1252	63.03%	70.57%	88.90%	85.25%
ADASYN	0.1281	61.50%	71.91%	88.47%	85.18%
Class Weight	0.1255	60.38%	72.93%	87.76%	84.82%
WGAN-GP	0.0681	80.81%	76.71%	95.45%	91.73%

**Figure 11:**
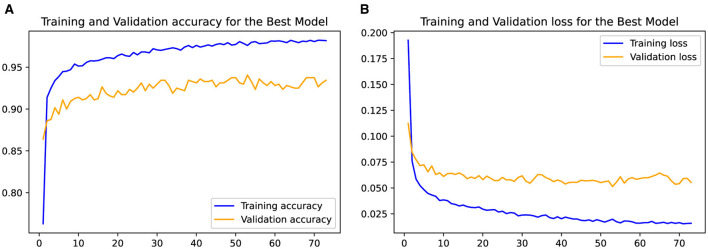
Evolution of the training and validation accuracy **(A)** and loss **(B)** as the LSTM model is trained for from 1 to 70 epochs.

In Figure 11, the plotted curves depict the training and validation accuracy and losses of the LSTM schemes employed for the seizure detection task. The training accuracy curve showcases the model's performance on the training data as the number of epochs increases, reflecting its learning progress. The ascending training accuracy indicates effective learning from the available data. Conversely, the validation accuracy curve illustrates the model's ability to generalize to unseen data. The decreasing training loss signifies convergence toward an optimal solution as the training progresses. Similar to the validation accuracy, the validation loss provides insight into the model's generalization to new data.

## 5 Conclusion

This study focused on seizure detection classification using a uni-directional LSTM model and explored the integration of generative data augmentation techniques. The findings provide valuable insights into the effectiveness of data augmentation approaches, with a particular emphasis on the use of GANs for addressing imbalanced datasets. The investigated GAN models include Vanilla GAN, CRAMER GAN, CGAN, and WGAN-GP, selected for their ability to generate realistic data distributions and improve the representation of minority classes. Additionally, conventional techniques such as SMOTE and ADASYN were also examined. Notably, the most significant improvements were observed with WGAN-GP, which demonstrated a strong alignment and resemblance to the real data distribution in projection analysis and QQ plots. This highlights the effectiveness of WGAN-GP in generating synthetic data that closely matches the real data distribution, thereby enhancing seizure detection performance and improving the classification accuracy of the LSTM classifier. The study serves as a foundation for future investigations and inspires further advancements in seizure detection research. However, additional research and evaluation are required to assess the generalizability of these findings across different datasets and compare the performance against other state-of-the-art approaches that incorporate data augmentation techniques.

## Data Availability

Publicly available datasets were analyzed in this study. This data can be found here: https://isip.piconepress.com/projects/nedc/html/tuh_eeg/#c_tusz.
